# A bacterial membrane-disrupting protein stimulates animal metamorphosis

**DOI:** 10.1128/mbio.03573-24

**Published:** 2024-12-27

**Authors:** Kyle E. Malter, Tiffany L. Dunbar, Carl Westin, Emily Darin, Josefa Rivera Alfaro, Nicholas J. Shikuma

**Affiliations:** 1 Department of Biology, San Diego State University, San Diego, California, USA; 2 Viral Information Institute, San Diego State University 7117 , San Diego, California, USA; Max Planck Institute for Marine Microbiology, Bremen, Germany

**Keywords:** metamorphosis, contractile injection system, effector, pore-forming toxin, cilia, toxin, secretion systems

## Abstract

Diverse marine animals undergo a metamorphic larval-to-juvenile transition in response to surface-bound bacteria. Although this host-microbe interaction is critical to establishing and maintaining marine animal populations, the functional activity of bacterial products and how they activate the host’s metamorphosis program has not yet been defined for any animal. The marine bacterium *Pseudoalteromonas luteoviolacea* stimulates the metamorphosis of a tubeworm called *Hydroides elegans* by producing a molecular syringe called metamorphosis-associated contractile structures (MACs). MACs stimulate metamorphosis by injecting a protein effector termed metamorphosis-inducing factor 1 (Mif1) into tubeworm larvae. Here, we show that MACs bind to tubeworm cilia and form visible pores on the cilia membrane surface, which are smaller and less numerous in the absence of Mif1. *In vitro*, Mif1 associates with eukaryotic lipid membranes and possesses phospholipase activity. MACs can also deliver Mif1 to human cell lines and cause parallel phenotypes, including cell surface binding, membrane disruption, calcium flux, and mitogen-activated protein kinase activation. Finally, MACs can also stimulate metamorphosis by delivering two unrelated membrane-disrupting proteins, MLKL and RegIIIɑ. Our findings demonstrate that membrane disruption by MACs and Mif1 is necessary for *Hydroides* metamorphosis, connecting the activity of a bacterial protein effector to the developmental transition of a marine animal.

**IMPORTANCE:**

This research describes a mechanism wherein a bacterium prompts the metamorphic development of an animal from larva to juvenile form by injecting a protein that disrupts membranes in the larval cilia. Specifically, results show that a bacterial contractile injection system and the protein effector it injects form pores in larval cilia, influencing critical signaling pathways like mitogen-activated protein kinase and calcium flux, ultimately driving animal metamorphosis. This discovery sheds light on how a bacterial protein effector exerts its activity through membrane disruption, a phenomenon observed in various bacterial toxins affecting cellular functions, and elicits a developmental response. This work reveals a potential strategy used by marine organisms to respond to microbial cues, which could inform efforts in coral reef restoration and biofouling prevention. The study’s insights into metamorphosis-associated contractile structures’ delivery of protein effectors to specific anatomical locations highlight prospects for future biomedical and environmental applications.

## INTRODUCTION

While microbes are often studied as pathogenic agents, many bacteria provide contextual and stimulatory information that promotes normal growth and development of eukaryotic or animal hosts (1–5). One instance in which microbes promote normal development is the stimulation of animal metamorphosis in response to bacteria. During these transient interactions in the sea, the free-swimming larvae of many marine invertebrates identify a suitable location for settlement and metamorphosis on the seafloor by sensing and responding to surface-bound bacteria (6, 7). This process of bacteria-stimulated metamorphosis is widespread among animals as diverse as corals, tubeworms, and urchins (8–11). Studying these interactions might, therefore, provide us with a fundamental understanding of the microbial products as well as host machinery that mediates, senses, and responds to these host-microbe encounters. While bacteria were first described to stimulate animal metamorphosis in the 1930s (12), the functional activity of bacterial products and how they activate the host’s metamorphic developmental program is not yet defined for any animal.

We have shown that the bacterium *Pseudoalteromonas luteoviolacea* stimulates the metamorphosis of the tubeworm *Hydroides elegans* by producing a phage tail-like structure called metamorphosis-associated contractile structures (MACs) (10, 13, 14). MACs are an example of a contractile injection system (*CIS*), which are related to the contractile tails of some bacterial viruses (phages) (15–17). *CISs* are composed of a rigid needle-like inner tube surrounded by a contractile sheath linked to a baseplate complex. CIS can be classified as type 6 secretion systems, which are bound to and act from the bacterial membrane, or extracellular *CIS* (eCIS), which assemble within the producing cell and are released extracellularly to autonomously interact with target cells. Upon binding to a target cell, contraction of the sheath propels the rigid inner tube through the target cell membrane, delivering a protein payload that elicits specific host responses in some cases (18–21).

Many *CISs* and the effectors they deliver mediate pathogenic interactions between the bacterium and host. For example, *CISs* elicit specific responses from target organisms by delivering protein effectors that possess cellular activities, including pore-formation and lipase activity (22, 23). However, *CISs* have also been shown to mediate beneficial interactions. For instance, a *CIS* produced by *Bradyrhizobium* promotes root nodule formation in legumes (24), and *CIS* promotes the intracellular survival of *Amoebophilus asiaticus* within its amoeba host (25). We have shown that MACs stimulate *Hydroides* metamorphosis by delivering a protein effector termed metamorphosis-inducing factor 1 (Mif1), which is both necessary and sufficient to induce *Hydroides* metamorphosis (18). Furthermore, Mif1 stimulates and requires diacylglycerol production, as well as protein kinase C (PKC) and mitogen-activated protein kinase (MAPK) signaling during *Hydroides* metamorphosis (26). However, how Mif1 functions and stimulates metamorphosis remains unclear. In this work, we set out to determine how the protein effector Mif1 from *P. luteoviolacea* MACs stimulates the metamorphosis of *Hydroides* larvae.

## RESULTS

### MACs form pores in *Hydroides* cilia and Mif1 enhances pore formation

**Fig 1:**
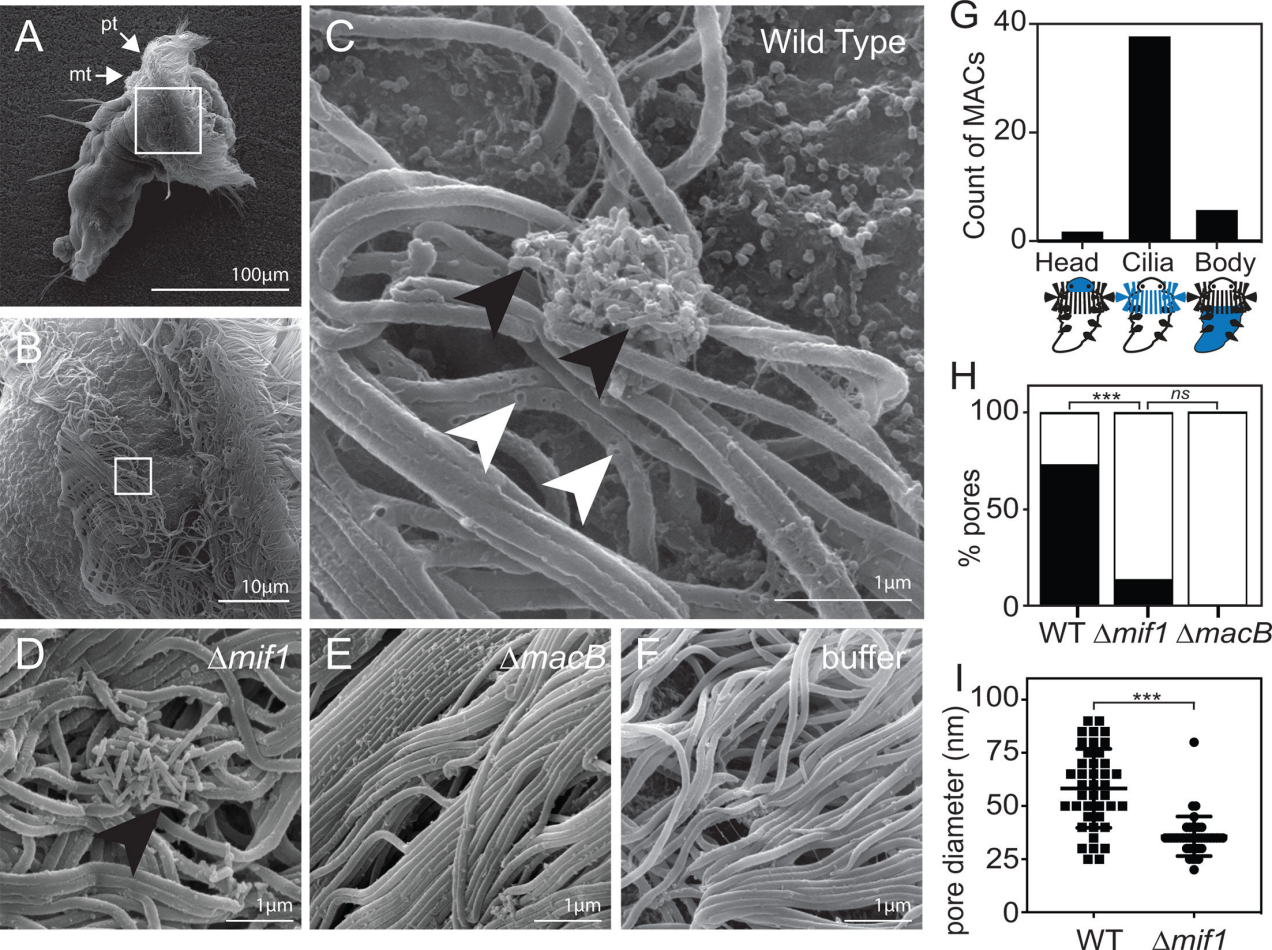
MACs form pores in *Hydroides* cilia, and Mif1 enhances pore formation. (**A**) Low-magnification view (400×) of *Hydroides* larvae exposed to WT MACs. pt, prototroch; mt, metatroch. White box indicates the location of magnification for panel B. (**B**) Mid-magnification view (3,200×) of *Hydroides* larval cilia exposed to WT MACs. White box indicates the location of magnification for panel C. (**C**) High-magnification view (23,500×) of *Hydroides* larval cilia exposed to WT MACs. Black arrows indicate MACs sheaths. White arrows indicate pores in cilia surrounding MACs array. (**D**) High-magnification view (25,000×) of *Hydroides* larval cilia exposed to ∆*mif1* MACs. (**E**) High-magnification view (15,000×) of *Hydroides* larval cilia exposed to ∆*macB* MACs. (**F**) High-magnification view (15,000×) of untreated *Hydroides* larval cilia. (**G**) Counts of MACs visualized on the head, cilia (prototroch or metatroch), or body of the larval animals. *N* = 46. (**H**) Counts of *Hydroides* larvae observed with WT, ∆*mif1,* and ∆*macB* MACs with cilia pores. *N* = 38, 26, and 15 larvae for WT, ∆*mif1,* and ∆*macB* exposures, respectively (***P < 1E-12; ns, not significant, two-tailed Student’s *t*-test, Bonferroni adjustment). (**I**) Diameters of pores observed on *Hydroides* larval cilia with WT and ∆*mif1* MACs. *N* = 41 for WT and ∆*mif1* (***P < 0.0001, two-tailed Student’s *t*-test).

While MACs have been shown to stimulate the metamorphosis of *Hydroides* larvae, the direct interaction between MACs and larval animals has not been previously observed. To observe this interaction, we performed scanning electron microscopy (SEM) on *Hydroides* larvae after exposure to MACs for 1 minute. We observed MACs bound to the larval prototroch or metatroch cilia (Fig. 1A through C, black arrows; Movie S1). As expected, we did not observe MACs when competent larvae were exposed to MACs’ preparations from a baseplate mutant (∆*macB*) or buffer control (Fig. 1E and F). Interestingly, MACs clusters displayed a higher propensity to bind to larval cilia rather than the body or head of the larvae (Fig. 1G). When viewing MACs at higher magnifications, individual cilia displayed numerous pores of varying diameters surrounding each MACs cluster (Fig. 1C, white arrows). In order to determine whether these pores were a result of MACs injection or Mif1 activity, we exposed *Hydroides* larvae to MACs that lacked the Mif1 payload (∆*mif1*). Larvae that were exposed to ∆*mif1* MACs bound to larvae at the same rate as wild-type MACs (4.22 MACs/larva) but displayed a significant decrease in the number of pores present surrounding the MACs’ clusters (Fig. 1C, D, and H; Fig. S1). The pore diameter caused by wild-type MACs was also larger than pores caused by MACs lacking Mif1 (Fig. 1I). These data suggest that MACs bind at high rates to the *Hydroides* larval cilia and cause the formation of pores that are larger and more numerous in the presence of Mif1.

### Mif1 binds to phosphoinositol lipids *in vitro* and causes membrane permeabilization

**Fig 2:**
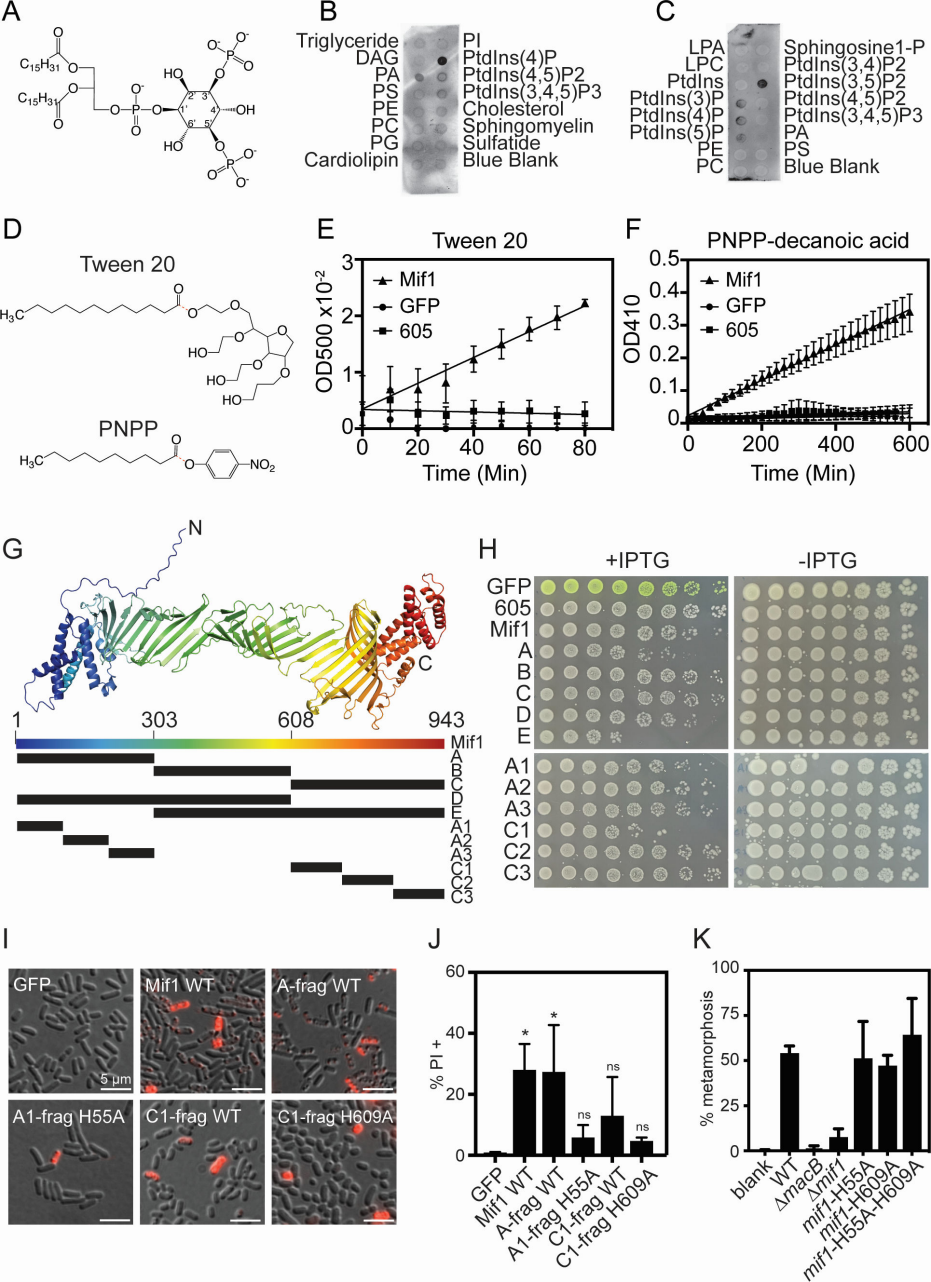
Mif1 binds to phosphoinositol lipids and possesses phospholipase activity *in vitro*. (**A**) Chemical structure of PtIns(3,5)P2. (**B and C**) Lipid-protein interaction assays were performed using purified Mif1 protein and Mif1 specific antibody on (**B**) a membrane lipid strip (Echelon Biosciences P-6002) or (**C**) a PIP strip membrane (Echelon Biosciences P-6001). (**D**) Chemical structure of 4-nitrophenyl phosphate (pNPP)-decanoic acid and Tween 20 esterase assay substrates. The chemical bond cleaved is indicated by a dotted red line. (**E**) Lipid cleavage assay with purified Mif1 protein, chaperone JF50_12605 (605) protein, or GFP protein incubated with Tween-20 in the presence of Ca^2+^ and liberated lauric acid was observed via turbidity. The average of four technical replicates is shown. (**F**) Esterase assay with purified Mif1, GFP, or chaperone JF50_12605(605) protein with decanoic acid-pNPP substrate. Cleavage and pNPP release occurs if acyl-ester linkage is hydrolyzed. Data are represented as the mean ± SD of *n* = 12 technical replicates across three independent biological replicates. (**G**) AlphaFold2 predicted Mif1 structural model and schematic representation of Mif1 fragments cloned for overexpression. Colors of the Mif1 model range from N- to C-terminus (blue-green-yellow-red) and are for orientation. (**H**) *Escherichia coli* expressing recombinant *mif1* (Mif1), *mif1* fragments, JF50_12605 (605), or *gfp* genes from an IPTG-inducible promoter in a pET15b vector in the presence or absence of 0.1 mM IPTG. Bacteria were grown overnight and then spotted by 1/5 serial dilutions starting at OD 1.0. (**I and J**) *E. coli* expressing recombinant full-length wild-type *mif1* (Mif1 WT)*, mif1* fragments, or *gfp* genes from an IPTG-inducible promoter in a pET15b vector after induction with 0.1 mM IPTG for 2 hours. (**I**) Representative images of cells after 15 minutes of incubation with propidium iodide (PI). Scale bar is 5 µm. (**J**) Bar graph quantifying the number of cells (%) stained with PI indicating cell permeabilization. Permeabilization is significantly different between GFP vs Mif1 WT or A-frag WT and not significantly different between GFP vs A1-frag H55A, C1-frag H609A, or C1-frag (*P < 0.05; ns, not significant, one-way ANOVA, Dunnett’s multiple comparisons test). Results shown are the average of three independent biological replicates; error bars are SD. (**K**) Comparison of blank control, WT, ∆*macB*, ∆*mif1, mif1*H55A, and *mif1*H609A mutation on the stimulation of *Hydroides* metamorphosis (***P < 0.001; ns, not significant, one-way ANOVA, Dunnett’s multiple comparisons test).

Because we observed that Mif1 enhances pore formation in cilia membranes, we tested whether recombinantly purified Mif1 is able to bind to lipids in commercially available phospholipid membrane strips (Fig. 2A). When exposed to the membrane strips, Mif1 bound to phosphatidylinositol-4-phosphate (Ptdins(4)P) with high affinity and, to a lesser degree, to Ptdins(4,5)P2, Ptdins(3,4,5)P3, and phosphatidic acid (Fig. 2B). Testing Mif1 binding against a second phosphatidylinositol lipid membrane showed that Mif1 had high affinity for phosphatidylinositol-3,5-phosphate (Ptdins(3,5)P2) and moderate affinity for Ptdins(3)P, Ptdins(4)P, and Ptdins(5)P (Fig. 2C). Our results show that full-length Mif1 preferentially binds several phosphatidylinositol lipids *in vitro*.

Many *CIS* effectors have been shown to possess phospholipase activity (23). When recombinantly purified Mif1 was exposed to a synthetic lipid decanoic acid-pNPP fusion or Tween-20 (Fig. 2D), we found that Mif1 possesses the ability to cleave the acyl-ester linkages of both substrates (Fig. 2E and F). Green fluorescence protein (GFP) or a putative MACs chaperone protein, JF50_12605, purified under the same conditions did not show esterase activity. These results show that Mif1 possesses esterase activity *in vitro*.

The Mif1 protein sequence does not match with significant homology to previously characterized proteins when run through the BLAST, HMMR, or Phyre2 protein prediction programs and protein databases (27, 28). However, a structural prediction of Mif1 (AlphaFold2, pLDDT = 59.8, pTM = 0.334, Fig. 2G) showed homology with a PapC translocation pore (PDB 2VQI, *E*-value 2.00E-50, %Identity = 31.5) and a BtuB outer membrane transporter (PDB 1NQE, *E*-value 1.00E-49, %Identity = 30.7) (29, 30), hinting that Mif1 might associate with the membrane of target cells.

To determine whether Mif1 causes the membrane permeabilization of intact cells, we cloned five fragments of Mif1 into an overexpression plasmid and expressed them in *Escherichia coli*. Mif1 fragments included the Mif1 protein divided into thirds (amino acids 1–304, 304–608, and 609–943, designated A, B, and C, respectively) and two larger portions covering two-thirds of the protein (amino acids 1–600 and 343–943, designated D and E, respectively) (Fig. 2G). Cells containing the full-length *mif1* and *mif1* fragments were serial diluted on media containing 0.1 mM IPTG. Growth inhibition was observed for cells expressing full-length *mif1* and fragments A, C, D, and E (Fig. 2H). Cells plated onto media lacking the IPTG inducer did not exhibit differences in growth when compared to a strain expressing *gfp*. To identify smaller active fragments of Mif1, we divided the A and C fragments into three overlapping proteins A1, A2, and A3 (amino acids 1–150, 50–200, and 151–304) and C1, C2, and C3 (amino acids 609–793, 693–843, and 793–943). Overproduction of the A1, A2, and C1 fragments showed moderate toxicity in *E. coli,* suggesting that the membrane disruption activity is located within the N- and C-termini of the Mif1 protein.

We next observed the loss of membrane integrity in *E. coli* overproducing Mif1 by exposing the cells to a membrane-impermeable stain propidium iodide (PI). Expression of the full Mif1 protein induced with IPTG in *E. coli* for 2 hours led to increased PI staining, while expression of GFP did not cause loss of membrane integrity (Fig. 2I and J). Similarly, expression of the Mif1-A and Mif1-C1 fragments led to increased PI staining. Some lipases possess catalytic serine, aspartate, and histidine residues (23), and single histidine residues were identified within the A (H55) and C1 (H609) fragments. Mutating each histidine to alanine was sufficient to abrogate PI staining and membrane disruption of the Mif1-A and -C1 fragments (Fig. 2I and J). In contrast, mutation of three aspartate residues (D686, D688, and D742) to alanine within the C1 fragment did not impact membrane permeability (Fig. S2). To determine whether the H55 or H609 residues are responsible for the activity of Mif1 to stimulate metamorphosis, we created *P. luteoviolacea* strains containing Mif1-H55A, Mif1-H609A, or Mif1-H55A-H609A mutations. When the Mif1 mutant strains were tested for their ability to stimulate *Hydroides* larval metamorphosis, we found that all three histidine mutants induced metamorphosis comparable to wild-type *mif1* (Fig. 2K). Our results demonstrate that Mif1 residues H55 and H609 lead to the loss of membrane integrity in *E. coli* but are not required for *Hydroides* metamorphosis.

### MACs target and permeabilize human HEK293T cells *in vitro*

**Fig 3:**
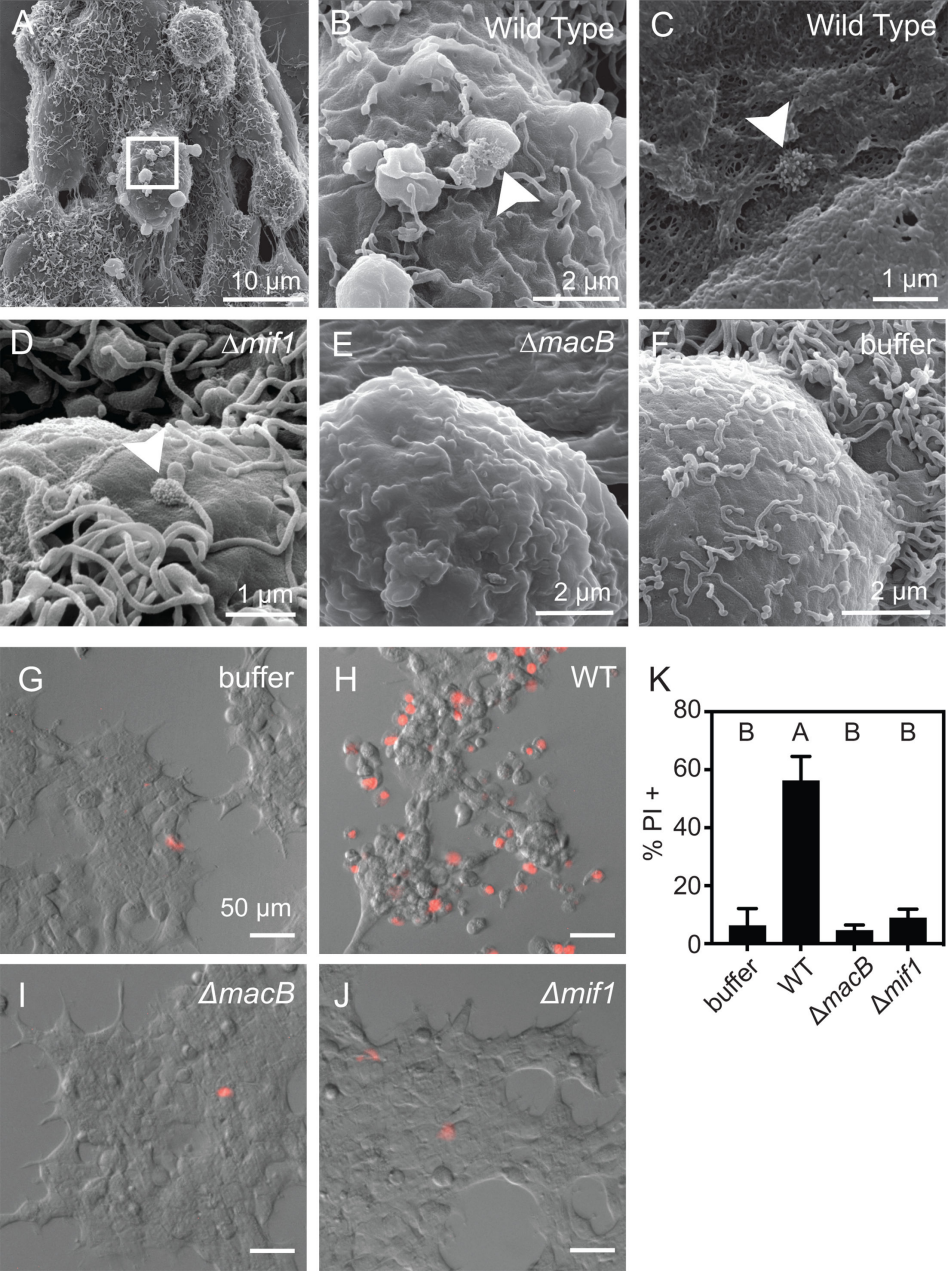
MACs bind to and permeabilize human tissue culture cells. (**A–F**) Scanning electron micrographs of human HEK293T cells exposed to MACs from (**A–C**) wild type, (**D**) ∆*mif1*, (**E**) ∆*macB,* or (**F**) buffer alone. MACs bound to HEK293T cells within 3 minutes of exposure (A: low magnification and B: high magnification). White box in panel A indicates the location of magnification for panel B. (**C**) MACs induce cell membrane degradation within 30 minutes. (**D**) HEK293T cells exposed to MACs from a ∆*mif1* mutant. Cell membrane degradation is reduced in the absence of *mif1*. (**B–D**) White arrows indicate the location of MACs array. (**G–J**) Representative images of PI-stained HEK293T cells after exposure to (**G**) buffer, (**H**) wild-type, (**I**) ∆*macB,* or (**J**) ∆*mif1* MACs. Scale bars are 50 µm. (**K**) Bar graph quantifying the number of cells (%) stained with PI indicating cell permeabilization. Permeabilization is significantly different between WT vs buffer, *P* = 0.0036; WT vs ∆*macB; P* = 0.0138; and WT vs ∆*mif1, P* = 0.0253; and not significantly different between buffer vs ∆*macB*, buffer vs ∆*mif1*, and ∆*macB* vs ∆*mif1* (one-way ANOVA, Tukey’s multiple comparisons test, letters represent Tukey *post hoc* test results). Results shown are the average of three independent biological replicates; error bars are SD.

While commercial molecular tools are not available for observing signal transduction in *Hydroides*, a number of tools are available for mammalian cell lines, and we have shown previously that MACs target eukaryotic cells (21). To develop an assay to study MACs and Mif1 activity with tissue culture cells, we tested whether human HEK293T cells were susceptible to MACs. To this end, we used SEM imaging and observed that MACs bound to the surface of HEK293T cells after 3 minutes of MACs exposure (Fig. 3A and B). After 30 minutes of MACs exposure, we observed the degradation of the HEK293T cell membrane (Fig. 3C). MACs’ mutants lacking the Mif1 effector (∆*mif1*) were still observed to bind to the cell surface; however, they did not induce membrane degradation (Fig. 3D). As expected, MACs were not observed on cells exposed to preparations from a ∆*macB* strain or buffer alone (Fig. 3E and F). To corroborate our observation of membrane degradation, we exposed HEK293T cells to MACs in the presence of a membrane-impermeant DNA stain (propidium iodide). We observed propidium iodide staining of 56% of HEK293T cells that were exposed to wild-type MACs (Fig. 3H and K). Similar to our observations with SEM imaging, the membrane permeabilization response was found to be dependent on the presence of Mif1 (∆*mif1*) (Fig. 3J and K). MACs’ preparations from a ∆*macB* strain or buffer alone exhibited low membrane permeabilization rates (Fig. 3G, I, and K). These results demonstrate that MACs bind to and permeabilize human HEK293T cells in a Mif1-dependent manner.

### MACs and Mif1 activate MAPK activity and calcium flux

**Fig 4:**
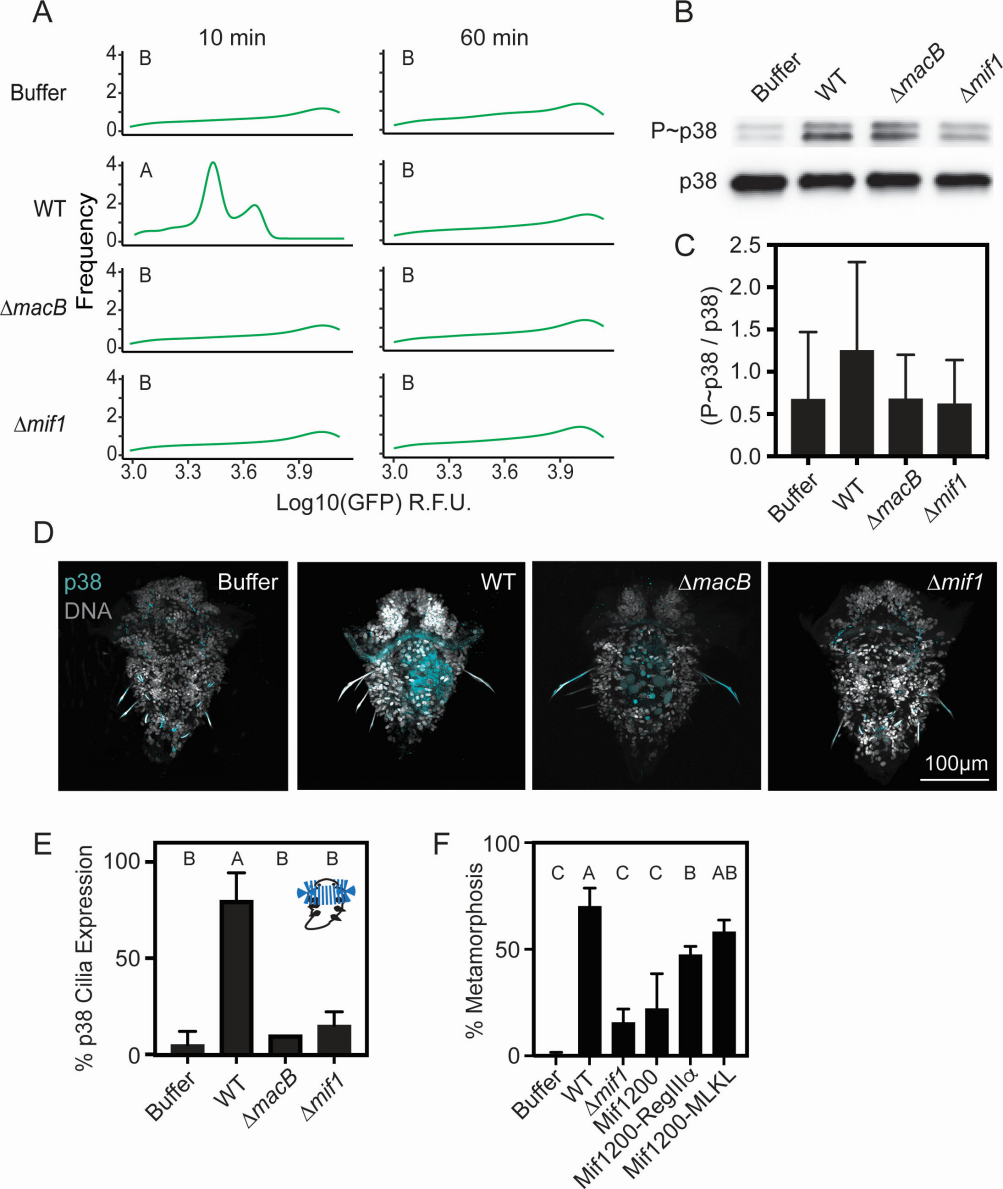
Mif1 activates MAPK activity and calcium flux. (**A**) Fluorescence intensity distribution of HEK293 cells transfected with a Ca^2+^ reporter after 10 minutes and 60 minutes of exposure to MACs’ extracts. GFP intensity values were transformed by log10. Mean intensity values were significantly different (*P* < 0.05, one-way ANOVA, Tukey *post hoc* test) between treatments after 10 minute exposure and were not significantly different after 1 hour exposure. (**B**) Western blot of HEK293T cells treated with MACs’ extracts for 5 minutes. Representative blots are shown for anti-phospho-p38 and total p38. (**C**) The ratio of phosphorylated p38 was compared to the total p38 (*n* = 3) (a one-way ANOVA with multiple comparisons showed WT vs ∆*macB P*-value = 0.80, WT vs buffer *P*-value = 0.79, and WT vs ∆*mif1 P*-value = 0.75). (**D**) Hybridizing chain reaction of p38 on larvae exposed to MACs’ extracts for 5 minutes from wild type, ∆*mif1*, ∆*macB,* or buffer alone. (**E**) Bar graph of percentage of larvae with ciliary band fluorescence (*n* = 10 per treatment averaged from two biological replicates). Letters represent one-way ANOVA and Tukey *post hoc* test results (*P* < 0.0001). (**F**) Graph of *Hydroides* percentage of metamorphosis in response to buffer, WT, ∆*mif1*, or MACs with Mif1-N200, Mif1-N200-RegIIIɑ, or Mif1-N200-MLKL. Letters represent one-way ANOVA and Tukey *post hoc* test results (Mif1-N200 vs Mif1-N200-RegIIIɑ =*P* < 0.03; Mif1-N200 vs Mif1-N200-MLKL = *P* < 0.001).

Many membrane-disrupting toxins have been shown to activate conserved signal transduction pathways in target eukaryotic cells, including calcium signaling and MAPK signaling (31–34). To test whether MACs activate calcium signaling, we transfected HEK293 cells with a plasmid expressing a genetically encoded calcium indicator (GCaMP6s) (35) and exposed the resulting cells to MACs. We observed that MACs caused an increase in intracellular calcium in the HEK293 cells after 10 minutes (Fig. 4A), and this calcium increase dissipated after 60 minutes of exposure to MACs. This calcium increase was not observed in cells exposed to MACs lacking Mif1 (∆*mif1*, Fig. 4A). Control exposures to ∆*macB* or buffer alone showed a minimal increase of calcium (Fig. 4A). Ionomyocin (10 mM) was used as a positive control, which matched the expression of WT MACs. Our results suggest that MACs cause an increase in intracellular calcium in a Mif1-dependent manner.

We have shown previously that *Hydroides* larvae upregulate p38 MAPK signaling in response to MACs (14). To test whether MACs and Mif1 activate MAPK signaling in human cell lines, we exposed HEK293T cells to MACs and performed western blot using a Thr180/Thyr182 phospho-p38 MAPK antibody. We observed that MACs activated p38 phosphorylation (Fig. 4B and C). However, phospho-p38 was reduced when cells were exposed to MACs lacking Mif1 (∆*mif1*). Because Mif1 activates p38 signaling in human cells, we asked whether the p38 expression in *Hydroides* was localized to a specific anatomical location. To this end, we performed hybridization chain reaction (HCR) on the *Hydroides* p38 MAPK gene. We found that the expression of p38 is consistently observed in the ciliary band of larvae induced with WT MACs’ extracts after 5 minutes of treatment exposure (Fig. 4D). In contrast, MACs lacking Mif1 (∆*mif1*) caused a lower activation of p38 expression, comparable to larvae treated with preparations from a ∆*macB* mutant or buffer alone. Quantification of the counts of larvae with p38 ciliary band expression corroborated the observations of larvae exposed to WT MACs compared to ∆*mif1, ∆macB,* or buffer treatments (Fig. 4E). These results demonstrate that MACs activate p38 phosphotransfer in human cells and p38 expression in *Hydroides* larval cilia in a Mif1-dependent manner.

If the metamorphosis response of *Hydroides* is a result of membrane permeabilization, we hypothesized that other membrane-permeabilizing proteins could substitute for Mif1 and stimulate metamorphosis. To test this hypothesis, we created two strains of *P. luteoviolacea* that possess the N-terminal 200 amino acids of Mif1 fused to two characterized membrane-permeabilizing proteins; RegIIIα or mixed lineage kinase domain-like protein (MLKL, oligomeric form T357E and S358D) (36–38). A third *P. luteoviolacea* strain was created that contains the N-terminal 200 amino acids of Mif1 alone as a control. When exposed to the MACs with modified payloads, *Hydroides* larvae underwent metamorphosis in response to MACs containing RegIIIα and MLKL (Fig. 4F). In contrast, MACs carrying only the N-terminal 200 amino acids of Mif1 did not significantly stimulate metamorphosis. These results suggest that *Hydroides* undergoes metamorphosis in response to membrane-permeabilizing proteins.

## DISCUSSION

**Fig 5:**
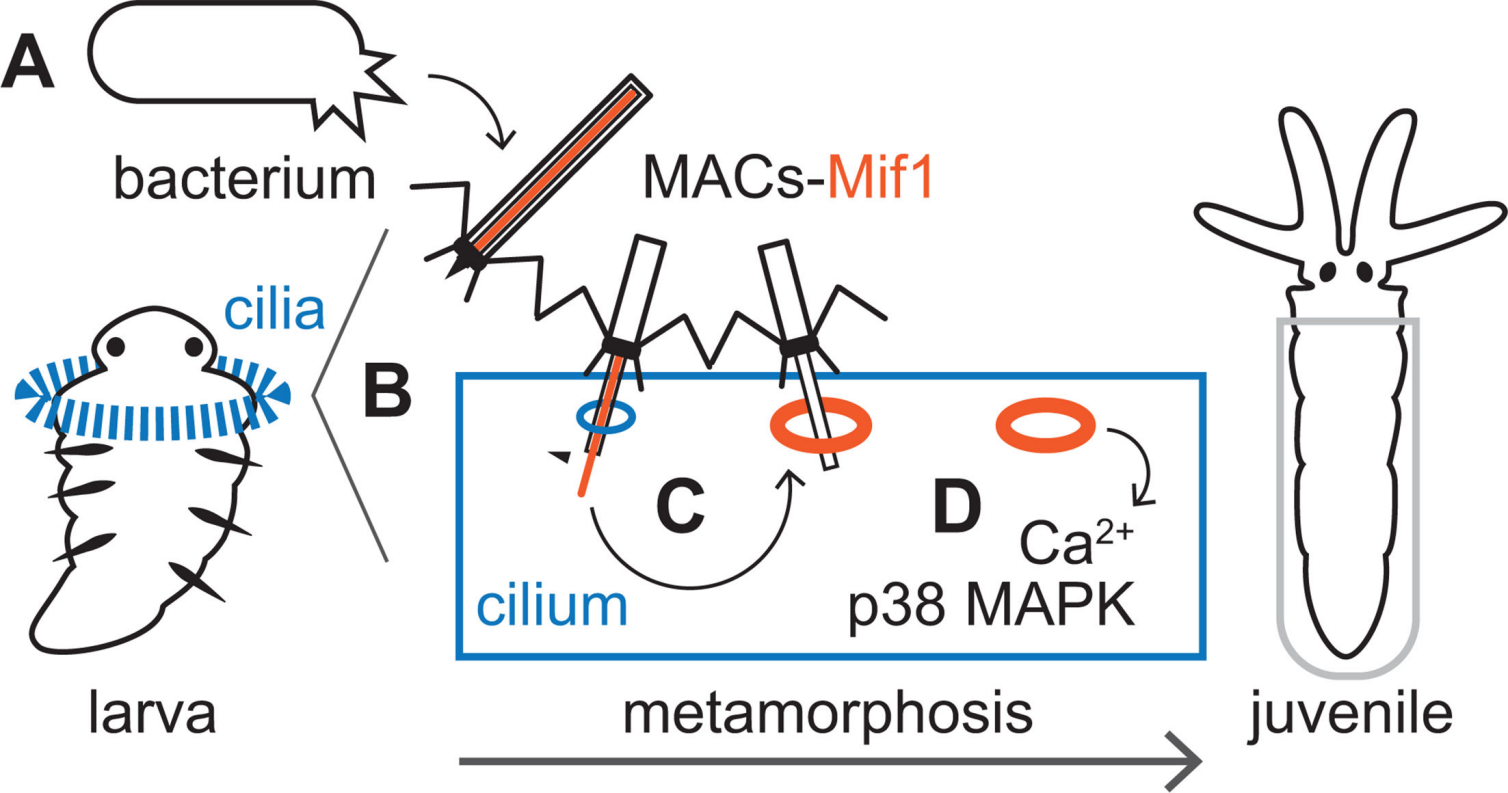
Model of *Hydroides* metamorphosis in response to MACs and Mif1. (**A**) *P. luteoviolacea* bacteria produce MACs loaded with the Mif1 payload, which are released via cell lysis. (**B**) *Hydroides* and MACs engage via the larvae’s ciliated band (blue). (**C**) Upon contact with *Hydroides* cilia, the MACs sheath contracts, driving the rigid inner tube through the cilia cell membrane. Subsequently, Mif1 (orange) located within the MACs inner tube lumen is delivered and promotes the number and size of pores. (**D**) Pore formation in the cilia, enhanced by Mif1, leads to calcium flux and phosphorylation of p38 MAPK, ultimately initiating the metamorphosis of the *Hydroides* larva to the juvenile form.

Here, we demonstrate that a bacterium stimulates the metamorphosis of an animal larva by injecting a membrane-disrupting protein into the cilia of the larval animal. Our results lead to the following mechanistic model (Fig. 5). The phage tail-like structures, MACs from *P. luteoviolacea* bacteria, are produced by cell lysis and carry Mif1 within the rigid needle-like tube (10, 18). MACs engage primarily with *Hydroides* larval cilia, puncture, and inject Mif1 into the cilia membranes, whereby Mif1 enhances the number and size of pores. The Mif1-enhanced pores lead to calcium flux, p38 MAPK signaling, PKC signaling, and, ultimately, metamorphosis (14, 26). These results have significant implications for our understanding of host-microbe biology and biotechnology as described below.

Many bacterial effectors exert their activity on target cells via lipase activity or membrane disruption (23, 39, 40). For example, the MARTX toxin from *Vibrio cholerae* binds to phosphatidylinositol lipids and, through phospholipase A1 cleavage, inhibits endosomal trafficking and autophagy (41). We show that MACs form visible pores in *Hydroides* larvae cilia and that pore formation is enhanced by Mif1. We cannot rule out the possibility that a *mif1* mutation causes pleiotropic effects on the MACs structure, leading to a reduction in pore number and size. However, we have previously demonstrated that a ∆*mif1* mutant forms intact MACs’ structures, as observed by cryo-electron tomography (18), suggesting that Mif1 is responsible for the enhanced pore formation. The relative size of pores observed on *Hydroides* cilia aligns with previous analyses of pores caused by cholesterol-dependent cytolysin pore-forming toxins (42). However, it is possible that fixation of the *Hydroides* larvae, MACs, and Mif1 distorted the pore size observed. The binding of Mif1 to specific phosphatidylinositol lipids *in vitro* and loss of membrane integrity upon expression in bacteria further support the role of Mif1 as a membrane-disrupting protein. The loss of membrane integrity caused by Mif1 may act independently of the phosphatidylinositol lipid-binding activity, as mutations of the H55 and H609 residues had no apparent effect on metamorphosis (Fig. 2). We also observed Mif1-dependent MACs binding, cell degradation, and cell permeabilization of HEK293T cells.

Second messenger signaling is critical for animals to undergo settlement and metamorphosis in response to bacteria (43–47). Previous work has shown that *Hydroides* relies on PKC, p38, and JNK (components of the MAPK signaling pathway) to undergo metamorphosis (14, 18, 26). The activation of the MAPK pathway has been demonstrated to be a conserved defense against pore-forming toxins, such as the Cry5B toxin in *Caenorhabditis elegans* and cytolysin in human macrophages (31, 32), and calcium flux has been linked with the activation of MAPK signaling (34). Consistent with these observations, we found that MACs activate p38 MAPK at the location of binding to *Hydroides* larval metatroch and prototroch cilia (Fig. 4).

The mechanisms of metamorphosis initiation in response to bacteria have remained mysterious. Calcium signaling has been implicated during bacteria-stimulated metamorphosis in hydrozoan larvae (48, 49) and polychaete larvae (50). In *Hydroides*, exposure to artificial inducers Cs+ and K+ induces metamorphosis, suggesting that membrane depolarization may be important for the stimulation of metamorphosis (51). However, membrane depolarization alone might not be sufficient for the stimulation of metamorphosis, as exposure to ionophores was shown to be insufficient for metamorphosis stimulation (51). Our observations that MACs and Mif1 bind to and cause pores in *Hydroides* cilia, activate p38 MAPK signaling, and cause calcium flux in human HEK293 cells provide a mechanistic explanation for this long-standing observation.

The anatomical location where animal larvae sense and respond to bacteria by undergoing settlement and metamorphosis has not yet been identified. Identifying the anatomical location of bacteria sensing could provide insight into the cell types and signal transduction mechanisms that mediate larval metamorphosis. In diverse host-microbe interactions, cilia often mediate the interactions between animal and bacterial cells. For example, a ciliated field on the light organ of the Hawaiian bobtail squid mediates the first contact between the squid and their *Aliivibrio fischeri* symbionts (52–54). Pneumolysin, a pore-forming toxin from *Streptococcus pneumoniae*, causes slowing of ciliary beat frequency in human epithelial cells (55). *Hydroides* larvae have been shown to slow their swimming speed in response to the presence of a bacterial biofilm or MACs (14, 56). Furthermore, previous work using laser ablation has ruled out the apical sensory organ as the receptor for settlement and metamorphosis in response to bacteria but implicated cilia on the ventral portions of the prototroch, metatroch, or food groove (57). Consistent with this hypothesis, we observed MACs bound in a majority of instances to the larval metatroch and prototroch cilia via SEM (Fig. 1) and activated p38 MAPK expression in a Mif1-dependent manner (Fig. 4). Determining whether MACs bind preferentially to specific cells or cell-surface markers is an open question and the subject of future work.

Effectors that cause membrane disruption are signatures of diverse microbial communities and can serve as an indicator of microbial-associated competitive activities (MACAs) (58). This hypothesis describes a phenomenon whereby eukaryotic cells sense MACAs as a strategy for coexisting with microbial communities and use them as information for decision-making about metabolism, growth, and development. For example, the proliferation of insulin-producing cells in zebrafish and mice is stimulated by the microbiome-derived membrane-disrupting protein BefA, which is hypothesized to induce host defenses that contribute to tissue development (59, 60). Similarly, *Hydroides* larvae may have co-opted the membrane-disrupting signature of MACs and Mif1, as a cue that promotes calcium flux, initiating MAPK signaling and leading to *Hydroides* metamorphosis. We observed a parallel upregulation of MAPK in human tissue cells, suggesting that *Hydroides’* response to Mif1 might be a conserved response to membrane disruption found in eukaryotic cells. Our result that RegIIIɑ and MLKL delivered via MACs also stimulate metamorphosis (Fig. 4F) suggests that membrane permeabilization may be a general mechanism of metamorphosis stimulation in *Hydroides*. We hypothesize that membrane disruption serves as a signature of microbial activity for animal larvae to sense and respond to surface-bound bacteria by undergoing settlement and metamorphosis. While the genes encoding MACs and Mif1 are present within only a subset of bacteria (15, 18, 19), bacterial molecules that disrupt membrane integrity are ubiquitous (61) and could provide a signature of bacterial activity and cue for animal larval metamorphosis. Understanding how different bacterial products stimulate metamorphosis could provide new strategies for promoting restoration efforts of environments like coral reefs or inhibiting processes such as biofouling (8).

Our insights into the function of MACs and Mif1 have significant implications for engineering *CIS* for future biomedical or biotechnology applications. An N-terminal signal peptide was recently described to load the protein effectors into the *Photorhabdus* virulence cassette (PVC) complex (62). Here, we show that MACs target and deliver a protein effector to human cell lines *in vitro*. We also demonstrate that MACs can be modified to deliver functional engineered payloads (Mif1-MLKL and Mif1-RegIIIɑ fusions). PVCs have recently been engineered to target specific cell surface markers (63), paving the way for using eCIS to target specific cell or tissue types. Our results that MACs deliver a protein payload to whole animals targeting a specific anatomical location (larval cilia) suggest that MACs have the potential to target specific tissue or cell types.

Our findings demonstrate the role of a bacterial membrane-disrupting protein and its functional abilities, which furthers our understanding of the complex nature of bacteria-animal interactions with implications for technology in restoration and biomedical fields.

## MATERIALS AND METHODS

### Cloning and strain generation

Standard gene cloning was performed as previously described (18, 64). Whole Plasmid Sequencing was performed by Plasmidsaurus using Oxford Nanopore Technology with custom analysis and annotation. Strains of *P. luteoviolacea* containing the specified in-frame deletions or point mutations were performed by double-homologous recombination as described previously (10, 18, 65).

### Recombinant protein expression and purification

*E. coli* BL21(DE3) pLysE containing the pET15b IPTG-inducible plasmid with the gene fragment of interest (*mif1*, *mif1* fragments, *gfp,* or JF50_12605) were grown shaking at 200 rpm and 37°C overnight. The overnight cultures were plated using a 1/5 serial dilution scheme with an initial OD_600_ of 0.1 and allowed to grow overnight at 37°C. The plates contained either LB with 100 µg/µL ampicillin with 0.1 mM IPTG or identical media lacking IPTG. BL21 plysE bacteria containing the pET15b plasmid expressing the individual bacteria constructs were struck out onto LB with ampicillin agar plates overnight. A single colony was inoculated into 5 mL of LB with ampicillin media culture tubes and grown overnight shaking at 200 rpm at 37°C. The entire 5 mL overnight cultures were added to 500 mL LB with ampicillin in beveled flasks shaking at 200 rpm at 37°C until reaching an OD_600_ of 0.4–1.0. The bacteria were induced with IPTG 0.1–1 mM, and the temperature was changed to 30°C during protein induction. Bacteria were allowed to grow for an additional 16 hours. Cell pellets were collected by centrifugation at 4,000 *g* for 20 minutes, and the supernatant was removed. Cell pellets were resuspended in 5 mL of 25 mM Tris pH 7.8, 500 mM NaCl, 20 mM imidazole, and reversible protease inhibitors, pepstatin A 20 µM, bestatin 10 µM, and leupeptin 100 µM. The samples were then stored resuspended at −80°C. After thawing, the samples were then lysed twice by French press with the Slm-Aminco French Press and mini cell at 1,000 psi. The collected lysates were then lysed by probe sonication for 30 seconds to disrupt DNA. The lysed samples were centrifuged at >10,000 *g* for 20 minutes, and the supernatant was collected and spun down for an additional 20 minutes. A volume of 2 mL of 70% slurry of Ni-NTA agarose beads was then equilibrated with the lysis buffer (25 mM Tris pH 7.8, 500 mM NaCl, and 20 mM imidazole) and added to the collected supernatants. The samples with Ni-NTA beads were allowed to rock at 4°C for at least 1 hour. The samples were spun down at 4,000 rpm for 5 minutes, and the unbound supernatant was removed and stored. The beads were then washed three times with 50 mL of ice-cold 25 mM Tris, pH 7.8, 500 mM NaCl, and 20 mM imidazole. The beads were then transferred to a separator column and incubated with 2 mL of elution buffer (25 mM Tris, pH 7.4, 500 mM NaCl, and 250 mM imidazole) for 15 minutes. The solution was then gravity filtered into a 15 mL conical tube. An additional 2 mL of elution buffer was gravity washed over the column, and the beads were spun down to remove the remaining buffer. The collected elution was buffer exchanged into 25 mM Tris, pH 7.6, 250 mM NaCl, and concentrated using pierce protein concentrators with a 10,000 Da molecular weight cutoff. Protein was then quantified by Bradford assay and stored at −80°C.

### Lipid binding assay

Membrane strips and PIP strips were obtained from Echelon biosciences (P-6001 and P-6002). The strips were blocked with 2.5% non-fat milk, 0.125% Tween-20, 1× PBS (137 mM NaCl, 2.7 mM KCl, 10 mM Na_2_HPO_4_, 1 mM CaCl_2_, and 0.5 mM MgCl_2_). A total of 3–5 µg of protein was added to the blocking buffer and left to rock at 4°C overnight. The strips were washed three times for 10 minutes with 1× PBST while rocking. A custom rabbit anti-Mif1 antibody (18) was added to the PBST at 1:1,000 dilution and rocked at 4°C overnight. The primary antibody was washed for 10 minutes with 1× PBST while rocking at room temperature. Finally, the secondary goat anti-rabbit antibody Thermo Fisher (31460) was added at 1:10,000 in 1× PBST and allowed to rock for 1 hour at room temperature. Three more washes with 1× PBST were completed for 15 minutes each prior to visualization with chemiluminescence.

### Tween-20 lipase assay

This protocol was modified from von Tigerstrom et al. (66). The lipase assay buffer consisted of 20 mM Tris-HCl, pH 8.0, 1.8% Tween-20, and 3 mM CaCl. The release and precipitation of the acyl chain were measured by turbidity at OD_500_. Twenty micrograms (1 µg/µL) of protein was added to 200 µL of assay buffer in a clear bottom 96-well plate and read at OD_500_ for 80 minutes. Protein activity was compared to control proteins GFP and JF50_12605 which were purified in tandem with Mif1.

### pNPP-decanoic acid assay

The pNPP-decanoic acid substrate was purchased from Sigma-Aldrich (N0252-100MG). The substrate solution was made with a final concentration of 50 mM Tris-HCl, pH 7.5, 1 mM CaCl_2_, 0.3% Triton X-100, 1 mM pNPP-decanoic acid, 4% (vol/vol) isopropanol, 1% (vol/vol) acetonitrile, and 15 µL protein at 1 µg/µL in 25 mM Tris, pH 7.6, and 250 mM NaCl. The reaction mixture was read at absorbance OD_405_ (Bio-Tek Synergy HT,) every 5 minutes for 10 hours.

### *E. coli* spotting assay

Bacteria were struck out onto LB agar plates with 100 µg/µL ampicillin and grown overnight at 37°C. A single colony was inoculated into 5 mL LB media with 100 µg/µL ampicillin and was shaken at 200 RPM at 37°C overnight. The cultures were then normalized by OD_600_ to an initial OD_600_ of 0.1 and serial diluted using a 1/5 serial dilution scheme (1, 1/5, 1/25, …etc.) for eight total serial dilutions. A volume of 5 µL of each dilution was spotted onto both a LB media plate containing 100 µg/µL ampicillin without IPTG and the same serial dilution was then plated onto a second plate containing LB with 100 µg/µL ampicillin and 0.1 mM IPTG. The cultures were grown overnight at 37°C and imaged after 22 hours of growth.

### *E. coli* membrane disruption and propidium iodide staining

*E. coli* containing the pET15b IPTG-inducible plasmid with gfp, full-length *mif1*, or *mif1* fragments were grown in LB media supplemented with 100 µg/µL ampicillin shaking at 200 RPM for 2 hours (OD_600nm_ = 0.6–1.1). The cultures were then induced with 0.1 mM IPTG and grown for two more hours. Cells were then pelleted at 4,000 *g* for 15 minutes, and the supernatant was removed. Cells were resuspended in LB. Propidium iodide (Invitrogen, P1304MP) was added to a final concentration of 1 µg/mL and incubated for 15 minutes before imaging.

### Microscopy

Microscopy was performed using a Zeiss Axio Observer.Z1 inverted microscope equipped with an Axiocam 506 mono camera and EC Plan-Neofluar 10×/0.3 Ph1/DICI (HEK293T cells) or Plan-Apochromat 100×/1.4 DICIII (bacterial cells) objectives. The Zeiss eGFP filter set 38 HE (excitation BP 470/40 [HE], beam splitter FT 495 [HE], and emission BP 525/50 [HE]) was used to capture calcium eGFP signal, and the Zeiss mRFP filter set 63 HE (excitation BP 572/25 [HE], beam splitter FT 590 [HE], and emission BP 629/62 [HE]) was used to capture propidium iodide staining. Images were captured using the same fluorescence exposure times across samples in the same replicate. ZEN 2 software was used for image processing. Bacterial cultures (3 µL) were added to 1% low-melt agarose (Apex Bioresearch Products, 20–103) pads on glass slides, and coverslips were placed on top. HEK293T cells were imaged in the multi-well plates they were grown in.

### Production of MACs extract

Purification of MACs was carried out as previously described (21). Briefly, from frozen stock, *Pseudoalteromonas luteoviolacea* HI1 and genetic derivatives were struck out to single colonies on to sea water tryptone (SWT) media (35.9 g/L Instant Ocean, 2.5 g/L tryptone, 1.5 g/L yeast extract, 1.5 mL/L glycerol, and 15 g/L agar for solid media) and grown overnight at 25°C. A single colony was inoculated into 5 mL SWT culture and grown overnight at 25°C and 200 rpm. The overnight culture was inoculated at 1:100 into a 50 mL flask and grown for 20–24 hours at 25°C and 200 rpm. The 50 mL overnight culture was then centrifuged at 4,800 *g* for 20 minutes at 4°C. The supernatant was removed, and the pellet was gently suspended in 5 mL of extraction buffer (20 mM Tris Base and 1 M NaCl, pH 7.5) with the aid of a serological pipette. The suspended pellet was then centrifuged again at 4,800 *g* for 20 minutes at 4°C. The supernatant was then removed and again centrifuged at 4,800 *g* for 20 minutes at 4°C to remove any remaining cells. After centrifugation, only the top 3 mL of supernatant was removed. MAC extract up to 2 days old was used for metamorphosis assays.

### Tissue culture

Human embryonic kidney cells (293T, ATCC CRL-3216) were cultured in tissue culture-treated flasks in DMEM, high glucose, HEPES (Gibco, 12430054) supplemented with 10% fetal bovine serum (FBS, Gibco, A3160401) at 37°C in 5% CO_2_. Human embryonic kidney cells (293 [HEK-293], ATCC 1573) were cultured in tissue-treated cell culture flasks in MEM (Gibco, 12430054) supplemented with 10% FBS.

### Tissue culture propidium iodide staining

HEK293T cells were seeded in 24-well tissue culture plates (Celltreat, 229213) and grown to 60%–70% confluency. MACs that were extracted no more than 1 week prior to treatment were added at a 1:10 ratio (e.g., 50 µL of extracts or elution buffer into 500 µL of cells) and incubated for 46–48 hours at 37°C in 5% CO_2_. Propidium iodide (1 µg/mL) was added and cells were incubated for 1 day before imaging. After imaging, growth media were transferred to a 1.5 mL tube. Cells were detached with 200 µL of 0.25% Trypsin-EDTA (Gibco, 25200056), neutralized with an equal volume of growth media, pipette mixed to disperse clumps, and transferred to the same tube the media was placed in. Cells were centrifuged at 4,000 *g* for 1 minute and then 100 *g* for 5 minutes. The supernatant was removed to approximately 100–200 µL, and cells were thoroughly resuspended. The percentage of PI-positive cells was quantified using a Countess 3 Automated Cell Counter (Invitrogen, AMQAX2000).

### Calcium flux assay

HEK293 cells were seeded in tissue culture treated 24-well plates (Celltreat, 229213) and transfected with pGP-CMV-GCaMP6s-CAAX (Addgene, 52228) using Lipofectamine Transfection Reagent (Invitrogen, 18324012) standard protocol. Cells were incubated at 37°C for 12 hours and then washed twice with calcium- and magnesium-free 1× PBS and trypsinized in 3 mL of 0.25% Trypsin-EDTA (Gibco, 25200056). Cells were seeded at 2E5 in 24-well plates in MEM with 0.5 mg/mL neomycin (MB Biomedicals, 180610). MEM with 0.5 mg/mL neomycin was replaced every 24 hours until cells reached 75% confluency. Once confluent, cells were exposed to a 1:10 dilution of MAC extract variants or buffer for 10 minutes and 1 hour. To induce calcium flux, 10 mM ionomycin (Sigma-Aldrich, 56092-81-0) was used as a positive control. Fluorescence was imaged in the GFP channel at 488 nm wavelength. Fluorescence intensity was measured by Zeiss Zen Program software and plotted as a histogram. Mean intensity values for each treatment at 10 minutes and 1 hour were compared by ANOVA and Tukey *post hoc* tests. This experiment was repeated three times to ensure reproducibility.

### Scanning electron microscopy of *Hydroides* larvae

Approximately 50 competent *Hydroides* larvae were placed into a 1.5 mL Eppendorf tube containing either 0.1 M sodium cacodylate buffer (Sigma-Aldrich, G5882) or filtered artificial saltwater (35 PSU). The larvae were exposed to purified MACs (WT, ∆*mif1*, and ∆*macB*) at a 1:200 dilution. The MACs were allowed to interact with the *Hydroides* larvae for 30 seconds before fixing the entire mixture in 2.5% glutaraldehyde (Sigma-Aldrich, G5882) overnight at 4°C. Following fixation, samples were loaded into a syringe and fixed onto a 0.2 µm membrane (Sigma-Aldrich, GTBP01300). The membranes were placed into separate wells of a 12-well culture plate for dehydration. Dehydration of samples was achieved by incubating the samples in increasing concentrations of ethanol. The concentrations were as follows: 30%, 50%, 70%, 80%, and 90%. The samples were then chemically dried using hexamethyldisilazane (Sigma-Aldrich, 379212). After chemically drying, the membranes were mounted onto 12.5 mm specimen stubs using 12.5 mm adhesive carbon tabs. Each membrane was then coated with 6 nm of platinum and imaged using a Quanta 450 FEG Scanning Electron Microscope. Scope parameters varied slightly from sample to sample but were in general as follows: 10 kV, working distance of 10–15 mm, 3.5 spot size, aperture 7, and 5° tilt.

### Scanning electron microscopy of HEK293T cells

HEK293T cells were grown directly on commercially available 0.2 µm membranes (Sigma-Aldrich, GTBP01300) and placed in individual wells of a 12-well cell culture plate (Costar, 3513). The cells were washed using three well volumes of DPBS (Gibco, 14040133). The cells were then exposed to purified MACs (WT, ∆*mif1*, and ∆*macB*) at a 1:10 and a 1:1 dilution. To visualize binding, the MACs were allowed to interact with the HEK293T cells for 3 minutes. To visualize cell permeabilization, MACs were allowed to interact for 30 minutes. Following each interaction time, the HEK293T and MACs mixtures were fixed in 2.5% glutaraldehyde (Sigma-Aldrich, G5882) diluted in 0.1 M sodium cacodylate (Sigma-Aldrich, 97068) overnight at 4°C. Following fixation, the fixative was washed away using 10 well volumes of DPBS. The cells were then dehydrated by incubating the membranes in increasing concentrations of ethanol. The concentrations were as follows: 50%, 70%, 80%, and 90%. The cells were then chemically dried using HMDS (Sigma-Aldrich, 379212). After chemically drying, the cells were mounted on 12.5 mm specimen stubs using 12.5 mm adhesive carbon tabs. Each stub was then coated with 6 nm of platinum and imaged using a Quanta 450 FEG Scanning Electron Microscope. Scope parameters varied, but general settings were similar to those used for *Hydroides* larvae samples.

### Tubeworm cultivation and metamorphosis assays

Specimens of *Hydroides elegans* were collected and maintained as previously described (13, 21, 26). Induction of *Hydroides* metamorphosis in response to bacterial strains was performed as previously described (26, 67).

### Western blot

HEK293T cells were seeded in 6-well tissue culture plates (Corning, 3516) and grown to 70%–80% confluency. MACs that were extracted no more than 1 day prior to treatment were added at a 1:75 ratio (e.g., 26.67 µL of extracts or elution buffer into 2 mL of cells) and incubated for 5 minutes before harvesting. Cells were lysed in 200 µL of RIPA buffer, 150 mM sodium chloride, 50 mM, pH 8.0 Tris-HCl, 1% Nonidet P-40, 0.5% sodium deoxycholate, and 0.1% SDS) and scraped with a cell scraper to remove cells from the plate. Protein lysate samples were normalized using Bradford protein assay (Bio-Rad, 5000006) measured at 595 nm on a plate reader. Equal concentrations of protein were loaded onto a stain-free SDS-PAGE gel 7.5% (Bio-Rad, 4568023). Protein loading was confirmed by stain-free gel imaging. Protein was transferred to a PVDF membrane. Themembrane was saturated with methanol and allowed to dry before cutting the membrane and incubating it with primary antibodies overnight at 4°C on a rocker. Primary antibody P-p38 MAPK rabbit mAb (Cell Signaling Technologies, 4511) or p38 MAPK rabbit mAb (Cell Signaling Technologies, 8690) was washed off the membrane in three washes with Tris-buffered saline with 0.1% Tween 20 detergent (TBST) for 10 minutes each. Secondary antibody (Goat anti-Rabbit IgG (H + L) Secondary Antibody, HRP, Invitrogen, 31460) was added at 1:20,000 in TBST and rocked for 1 hour at room temperature. The membrane was washed four times for 15 minutes each with TBST before imaging. Imaging was performed using a chemiluminescent substrate (SuperSignal West Femto Maximum Sensitivity Substrate, Thermo Scientific, 34095) and acquired using Azure 280 imaging system (Azure Biosystems).

### Hybridization chain reaction

Whole mount embryo HCRs were carried out as described in reference (68) in 1.5 mL tubes. Probes were designed by Molecular Instruments using mRNA coding sequences from *Hydroides* transcriptome. *Hydroides* probes were designed based on genes p38 (XLOC_080548) and FOS (XLOC_080516). Amplifiers and probe-washing buffers were purchased from Molecular Instruments. Larvae were exposed to a 1:100 dilution of MACs for 5 and 30 minutes. Larvae were then treated in 6.5% MgCl to relax larvae and then washed twice with FSW. Larvae were fixed in 4% PFA (2.5 mL 16% PFA, 1 mL 10× PBS Buffer, and 6.5 mL RNase-free H_2_O) overnight at 4°C on a nutator. Samples were washed in 5× SSCT (10 mL of 20× sodium citrate, 400 µL 10% Tween 20, and 44.5 mL of RNase-free H_2_O), and probes were added to hybridization buffer at a concentration of 1 pM per gene. Samples were pre-hybridized for 30 minutes at 37°C before probes were added. The following day, samples were washed four times in intervals of 30 minutes with probe wash buffer. Hairpins were heat shocked at 95°C for 90 seconds and snap cooled for 30 minutes at room temperature. Hairpins and amplifier buffer were added to samples and incubated overnight at 37°C. The following day, samples were washed four times with 5× SSCT in 30 minute intervals and mounted in ProLong Antifade Diamond Mountant with DAPI (P36961). Samples were imaged the same day on a Zeiss Axio Observer.Z1 inverted microscope equipped with an Axiocam 506 mono camera and Neofluar10x/0.3 Ph1 20× objective. Hairpins for p38 were AlexaFluor 594 and AlexaFluor 647 for FOS.

## SUPPLEMENTAL MATERIAL

Supplemental Material mbio.03573-24-s0001.docx Supplemental tables and figures; legend to Movie S1.

Movie S1 mbio.03573-24-s0002.mp4 MACs bound to *Hydroides* larval cilia.

ASM does not own the copyrights to Supplemental Material that may be linked to, or accessed through, an article. The authors have granted ASM a non-exclusive, world-wide license to publish the Supplemental Material files. Please contact the corresponding author directly for reuse.
