# Supplementary material for: A bacterial membrane-disrupting protein stimulates animal metamorphosis
Source: mBio. 2024 Dec 27;16(2):e03573-24. doi: 10.1128/mbio.03573-24 (PMC11796346; doi:10.1128/mbio.03573-24)
Supplement: Supplemental Material — Supplemental tables and figures; legend to Movie S1. [file mbio.03573-24-s0001.docx]

**SUPPLEMENTARY MATERIAL**

**TITLE**

**A Bacterial Membrane-Disrupting Protein Stimulates Animal Metamorphosis**

**AUTHORS**

Kyle E. Malter*, Tiffany L. Dunbar*, Carl Westin, Emily Darin, Josefa Rivera Alfaro, and

Nicholas J. Shikuma#

Department of Biology and Viral Information Institute, San Diego State University, San Diego, California 92182 USA

* Co-first authors

# Corresponding author: Nicholas J. Shikuma ([nshikuma@sdsu.edu](mailto:nshikuma@sdsu.edu))

**Table S1. Strains used in this study.**

| **Strain no.** | **Strain** | **Genotype** | **Source** |
| --- | --- | --- | --- |
| NJS005 | *Pseudoalteromonas luteoviolacea* HI1 | StrR | (Huang et al., 2012) |
| NJS023 | *Pseudoalteromonas luteoviolacea* HI1 | StrR, ∆*macB* | (Shikuma et al., 2014) |
| NJS279 | *Pseudoalteromonas luteoviolacea* HI1 | StrR, ∆*mif1* | (Ericson et al., 2019) |
| NJS213 | *Pseudoalteromonas luteoviolacea* HI1 | StrR, ∆*macB* | (Shikuma et al., 2014) |
| NJS696 | *Pseudoalteromonas luteoviolacea* HI1 | StrR, *mif1 H55A* | This study |
| NJS787 | *Pseudoalteromonas luteoviolacea* HI1 | StrR, *mif1 H609A* | This study |
| NJS836 | *Pseudoalteromonas luteoviolacea* HI1 | StrR, *mif1 H55A H609A* | This study |
| NJS876 | *Pseudoalteromonas luteoviolacea* HI1 | StrR, *mif1-*N200 | This study |
| NJS872 | *Pseudoalteromonas luteoviolacea* HI1 | StrR, *mif1*-N200-RegIIIɑ | This study |
| NJS873 | *Pseudoalteromonas luteoviolacea* HI1 | StrR, *mif1-*N200-MLKL | This study |

**Table S2. Plasmids used in this study.**

| **No.** | **Plasmid** | **Genotype** | **Source** |
| --- | --- | --- | --- |
| pNJS007 | pCVD443 | Amp^R^, Km^R^, sacB, pGP704 derivative | (Huang et al., 2012) |
| pNJS035 | pCVD443∆*macB* | pCVD443::∆*macB* Amp^R^ , Km^R^ | (Shikuma et al., 2014) |
| pNJS261 | pCVD443∆*mif1* | pCVD443::∆*mif1* Amp^R^ , Km^R^ | This Study |
| pNJS397 | pET15b-GFP | Amp^R^, GFP | (Ericson et al., 2019) |
| pNJS393 | pET15b-*605* | Amp^R^, 605 | (Ericson et al., 2019) |
| pNJS395 | pET15b-*mif1* | Amp^R^, Mif1 | (Ericson et al., 2019) |
| pNJS505 | pET15b-*mif1* 1-304 | Amp^R^, A | This Study |
| pNJS506 | pET15b-*mif1* 304-608 | Amp^R^, B | This Study |
| pNJS507 | pET15b-*mif1* 609-943 | Amp^R^, C | This Study |
| pNJS423 | pET15b-*mif1* 1-600 | Amp^R^, D | This Study |
| pNJS424 | pET15b-*mif1* 343-934 | Amp^R^, E | This Study |
| pNJS729 | pET15b-*mif1* 1-150 | Amp^R^, A1 | This Study |
| pNJS730 | pET15b-*mif1* 50-200 | Amp^R^, A2 | This Study |
| pNJS756 | pET15b-*mif1* 151-304 | Amp^R^, A3 | This Study |
| pNJS742 | pET15b-*mif1* 609-793 | Amp^R^, C1 | This Study |
| pNJS743 | pET15b-*mif1* 693-843 | Amp^R^, C2 | This Study |
| pNJS744 | pET15b-*mif1* 793-943 | Amp^R^, C3 | This Study |
| pNJS787 | pCVD443_Nif1_H55A | pCVD443::Mif1-H55A Amp^R^, Km^R^ | This Study |
| pNJS956 | pCVD443_Nif1_H609A | pCVD443::Mif1-H609A Amp^R^, Km^R^ | This Study |
| pNJS1105 | pCVD443_Mif1-N200 | pCVD443::Mif1-N200 | This Study |
| pNJS1114 | pCVD443_Mif1-N200-regIIIalpha | pCVD443::Mif1-N200-regIIIalpha | This Study |
| pNJS1115 | pCVD443_Mif1-N200-MLKL | pCVD443::Mif1-N200-MLKL | This Study |
| pNJS1019 | pGP-CMV-GCaMP6s-CAAX | Calcium indicator (GCaMP6s) | (Tsai et al., 2014) |
|  |  |  |  |


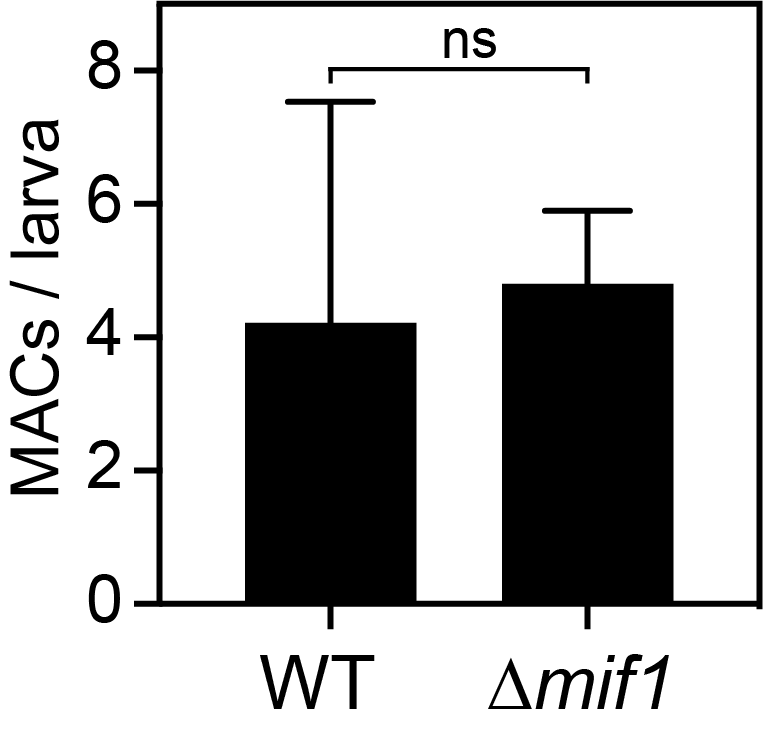


**Figure S1. MACs per larva.** Bar graph quantifying the number of MACs arrays observed on each larva via SEM. Bars indicate averages, error bars indicate standard deviation. N = 9 WT, N = 5 ∆*mif1*. Two-tailed Mann-Whitney test, P = 0.2977. ns = not significant.

**
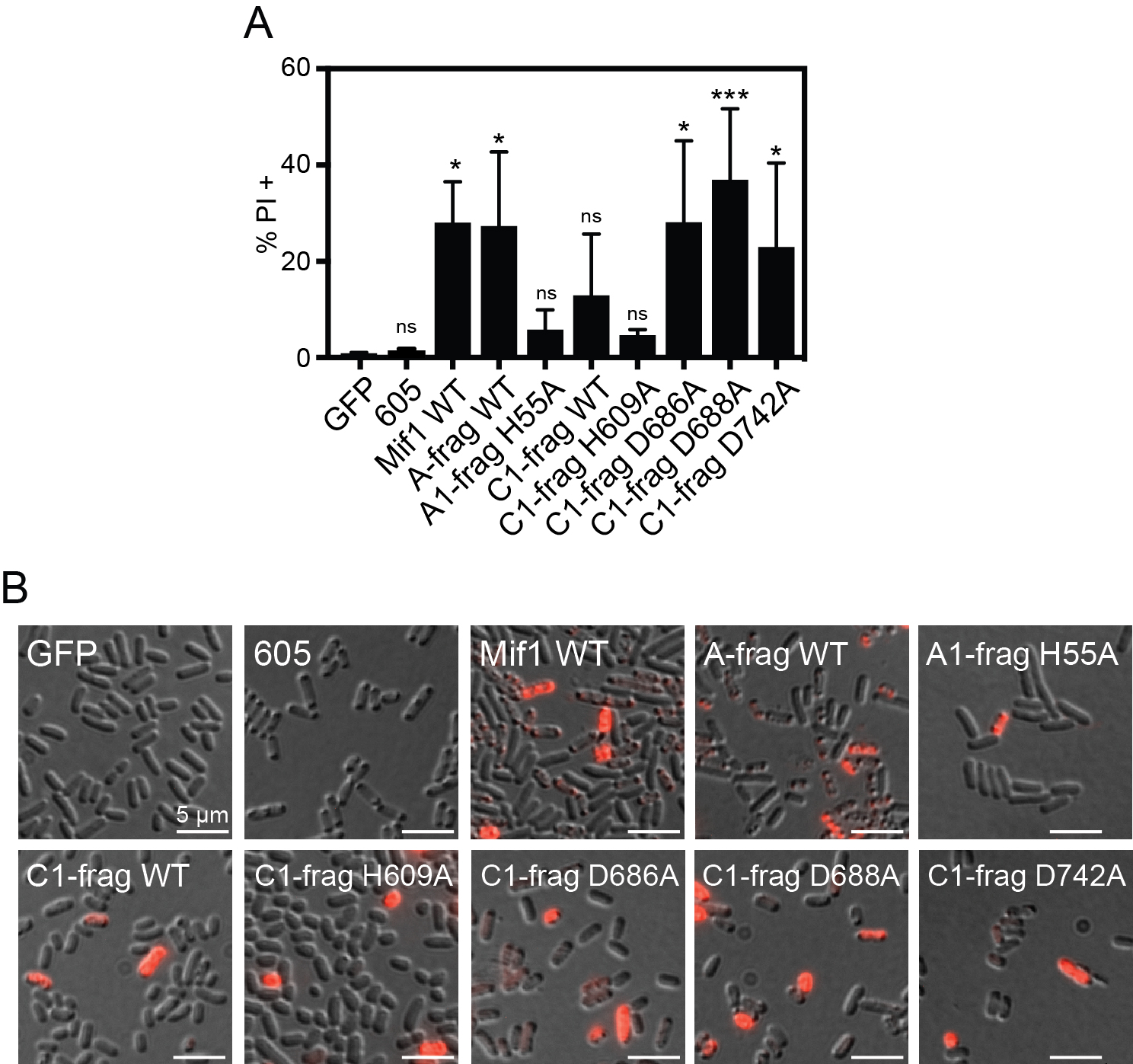
**

**Figure S2. Heterologous expression of protein in *E. coli* and membrane permeability.** (A) Bar graph quantifying the number of cells (%) stained with propidium iodide (PI) indicating cell permeabilization. Permeabilization is significantly different between GFP vs. Mif1 WT, A-frag WT, C1-frag D686A, or vs. C1-frag D688A, and not significantly different between GFP vs. 605, A1-frag H55A, C1-frag H609A, or vs. C1-frag WT (*** = p < 0.001, * = p < 0.05, ns = not significant, One-way ANOVA, Dunnett’s multiple comparisons test). Results shown are the average of three independent biological replicates, error bars are SD. (BA) Representative images of cells after 15 minutes of incubation with propidium iodide. Scale bar is 5 µm.

**Movie S1. MACs bound to *Hydroides* larval cilia.** Low-magnification view (400x) to high-magnification view (23,500x) of *Hydroides* larval ciliary band (metatroch) exposed to WT MACs.
